# Winter bird-window collisions: mitigation success, risk factors, and implementation challenges

**DOI:** 10.7717/peerj.7620

**Published:** 2019-09-02

**Authors:** Barbara B. Brown, Erika Kusakabe, Angelo Antonopoulos, Sarah Siddoway, Lisa Thompson

**Affiliations:** 1 Family & Consumer Studies, University of Utah, Salt Lake City, UT, United States of America; 2 Natural History Museum of Utah, University of Utah, Salt Lake City, UT, United States of America

**Keywords:** Bird, Window collisions/strikes, Prevention, Mitigation, Citizen science, Reflective windows, Fruiting trees, Bird-friendly window film, Cedar waxwings

## Abstract

Millions of birds die in bird-window collisions in the United States each year. In specialized test settings, researchers have developed methods to alter window designs to mitigate collisions. However, few published studies provide pretest and posttest evaluations of mitigation treatment areas and untreated control areas on existing buildings. We initially monitored bird-window collisions at a single building on the University of Utah campus in Salt Lake City, Utah, USA, during winter 1 (November 9, 2017–January 2, 2018). We found 15 bird-window collisions, most under a portion of the building with a mirrored façade. To test a mitigation treatment, we installed Feather Friendly® bird deterrent film on part of the mirrored façade after winter 1. The unmitigated areas of the same building served as a control area. We continued monitoring during the following winter 2 (November 15, 2018–January 12, 2019). The treated area collisions declined from seven before mitigation to two after mitigation, a 71% reduction. The control area had eight collisions at both times. Results of a generalized estimating equation yielded a significant area by season interaction effect (*p* = 0.03) and fewer collisions in the mitigated area than the control area at winter 2 (*p* = 0.03), supporting efficacy of the mitigation. In winter 2 we also expanded monitoring to eight total buildings to evaluate the risks of mirrored windows and proximity to fruiting pear trees (*Prunus calleryana*) and the benefits of bird-friendly glass. Bird-friendly glass, found on two buildings, included windows with permanent fritted dots or embedded ultraviolet patterns. We counted 22 collisions across the eight buildings. Mirrored windows and proximity to fruiting pear trees related to higher odds of bird-window collisions, based on separate generalized estimating equations. The best fit model included mirrored windows and pear trees. The two buildings with bird-friendly glass had only one collision, suggesting that these designs deter collisions, although the difference was not statistically significant. To publicize the study and to receive reports of additional bird collisions or fatalities on campus, we created a citizen science project on iNaturalist and engaged in additional outreach efforts that yielded 22 ad hoc reports. Many previous studies have documented Cedar Waxwing (*Bombycilla cedrorum*) collisions, but at relatively low numbers. Cedar Waxwings accounted for 31 of 34 identifiable collisions from the monitoring study and 4 of 21 identifiable collisions or fatalities from ad hoc reports.

## Introduction

Between 365 million and 988 million birds die in the U.S. each year due to bird collisions against buildings, especially buildings with reflective windows, based on estimates from a systematic review of 23 studies (Loss et al., 2014). Bird-window collision studies often identify collision risk factors, but rarely result in mitigation (Schneider et al., 2018). In November 2017, incidental observation of a non-fatal Cedar Waxwing window collision and documentation of subsequent collisions motivated the mitigation of one building at the University of Utah. We evaluated mitigation efficacy in conjunction with tests of collision risk factors across campus during winter, which is consistent with the call to study collisions outside of migratory seasons (Hager et al., 2017).

Most mitigation studies have established evidence of effective bird-deterrent window treatments by using one of two experimental protocols. First, in specially designed flight tunnels or cages, captured birds will fly less often toward effectively mitigated windows than toward untreated control windows (Klem, 2009; Klem & Saenger, 2013; Rössler, Nemeth & Bruckner, 2015). Second, for free-hanging windows suspended near a woodland edge, wild birds will also fly less often toward effectively mitigated than control windows (Klem, 1989; Klem, 1990; Klem, 2009). These experiments have shown that effective mitigation typically requires exterior window applications, such as stripes or dots, to be applied densely, at most 10 cm apart vertically by five cm horizontally (Klem, 2008; Klem, 2009). However, researchers need to test mitigation efficacy beyond flight tunnels and free-hanging windows. Buildings in the landscape each offer their own “mortality signatures,” with substantial variability in bird fatalities from one building to the next (Hager et al., 2013). This variability requires more tests of mitigation and collision risk factors across diverse buildings, landscapes, species, and seasons.

The dearth of research on mitigation of existing buildings may reflect the difficulties of achieving strong quasi-experimental study designs (Cook & Campbell, 1979) or before-after control-impact designs (Underwood, 1992) in such settings. These designs require pretest baseline data collection in a treatment area and a control area, then mitigation of the treatment area, then posttest data collection from both areas in the same season as baseline measurement. Control areas with unmitigated windows are key to establishing that mitigation is the cause of any decreased collisions (Cook & Campbell, 1979), instead of other factors, such as yearly fluctuations in the presence of nomadic Cedar Waxwings (Kaufman, 2001). To date, several mitigation implementations have shown reduced collisions (Brisque, Campos-Silva & Piratelli, 2017; Durrance, 2015; Ocampo-Peñuela et al., 2016b; Winton, Ocampo-Peñuela & Cagle, 2018). However, these studies have not included all three stringent evaluation elements: control groups, similar seasons in pretest and posttest, and statistical tests that evaluate area (treatment vs. control) and season (pretest vs. posttest winters) and their interaction. Our study fills a gap by conducting a mitigation evaluation with all three key design and analysis components needed to strengthen our confidence in mitigation effects.

In the second part of our study, we evaluated three collision risk factors and included a campus outreach component to raise awareness and identify additional bird collision or fatality problems. Previous studies found that the number of bird-window collisions increased with greater areas of glass, higher percentages of glass in façades, or more continuous surfaces of glass (Borden et al., 2010; Hager et al., 2013; Kahle, Flannery & Dumbacher, 2016; Klem et al., 2009; Ocampo-Peñuela et al., 2016b). Some technical reports or articles indicated high risks for mirrored windows (Evans Ogden, 2002; O’Connell, 2001; Sheppard & Phillips, 2015), but without explicit comparisons between mirrored and other types of glass. We examine whether mirrored windows pose a risk factor. Past research has also demonstrated that nearby trees or tree reflections in windows relate to window collision risk (Borden et al., 2010; Gelb & Delacretaz, 2006; Klem et al., 2009; Kummer, Bayne & Machtans, 2016b). We focused more specifically on risks related to fruiting pear trees. Our initial observation of a window collision involved a Cedar Waxwing, a species that depends on winter fruit for food (Witmer, Mountjoy & Elliot, 2014), and waxwing flocks had been observed using the many local pear trees. Finally, we tested whether buildings with bird-friendly glass, defined as glass windows with permanent frits or UV patterns intended to deter collisions, experienced reduced collision risks.

In sum, we asked whether mitigation was effective at a high-collision site. We also tested whether mirrored windows and nearby fruiting pear trees were associated with greater collision risks while buildings with bird-friendly glass were associated with lower collision risks. Finally, we assessed whether campus outreach uncovered other dangerous places on campus for birds.

## Methods

### Study site

The University of Utah is located on the eastern edge of Salt Lake City, UT. Designated as a state arboretum, the campus has over 12,000 trees (Robles, 2018) and 77 buildings on main campus. Many of the trees provide cover and food for birds. Examples include ornamental pear trees (*Pyrus calleryana*), flowering crabapples (multiple *Malus* species), blue spruce (*Picea pungens*), white fir (*Abies concolor*), English hawthorn (*Crataegus laevigata*), and Gambel oak (*Quercus gambellii*).

### Mitigation applied to one building

**Figure 1:**
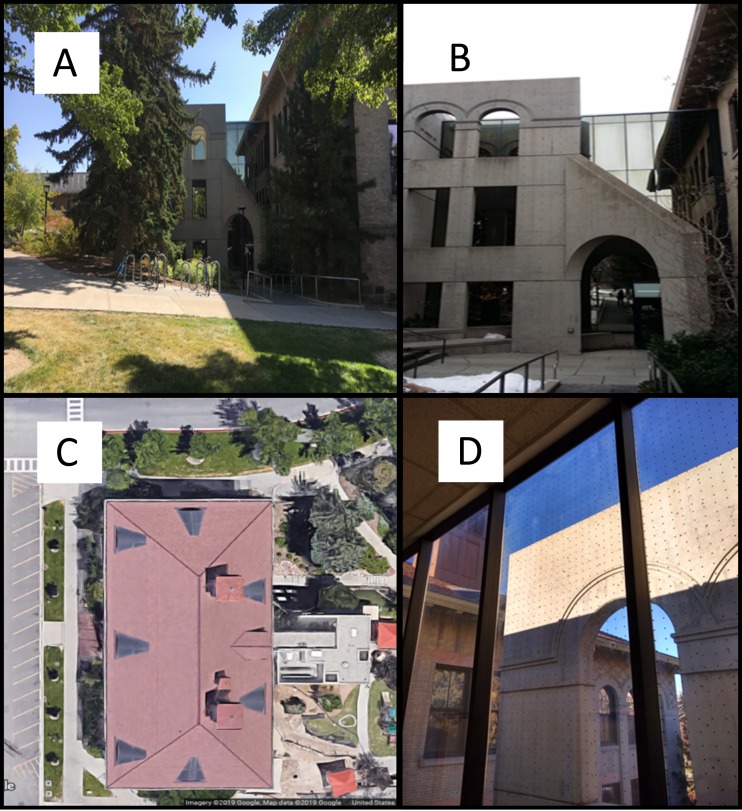
Views of the building that received bird-window collision mitigation. (A) Mitigation building extension pictured between limbs of closest pear trees. (B) False wall partially covers view of mirrored wall 2.3 m away. (C) Aerial view of historic red-roofed building shows small grey-roofed addition on right side of building that has the mirrored façade (D) View from interior third floor shows mitigation dot pattern applied to exterior mirror. Photo credit: Barbara B. Brown. Map data ©2019 Google.

What we subsequently call the “mitigation building” is a 1901 3-story structure, with reflective windows on the historic part of the building. An eastern wing, added in the mid-1980s (Fig. 1C), is clad in continuous mirrored panels on its north face (Fig. 1A). A non-university firm erected scaffolding to access the façade and applied Feather Friendly^®^ bird deterrent film to the mirror’s exterior on November 13, 2018. They then removed the film backing, leaving small white dots, spaced five cm apart. The budget was sufficient to allow 37 square meters of coverage, which they applied to the top two stories of the eastern side. The ground level had a small maple tree (*Acer*) in a planting box blocking part of the first floor façade. The resulting view through the mitigated window is mostly clear. The western side served as the control area. Unlike the experimental settings of flight tunnels or free-hanging windows, this building has a false wall that partially blocks views of the mirrored façade, which might pose difficulties for birds (Figs. 1B and 1D).

### Collisions risk factors for eight buildings

After mitigation, we monitored winter bird collisions at eight buildings, including the mitigation building. The buildings were chosen to assess effects of three risk factors for bird-window collisions. The mitigation building had the risk factor of a mirrored surface. Three buildings had the risk factor of being within 4.9 m of mature fruiting ornamental pear trees. We focused on pear trees because we observed many waxwings spending substantial time feeding from the numerous pear trees near the mitigation building and they are an important winter food source (Witmer, 1996; Witmer, Mountjoy & Elliot, 2014). Two buildings had the low-risk factor of windows with bird-friendly glass that had permanent visible ceramic frits or embedded ORNILUX Mikado UV patterns. Fritted glass, often chosen to reduce heat loss and preserve views, also deters bird strikes (Klem, 2009; Ocampo-Peñuela et al., 2016b). ORNILUX Mikado windows, with embedded UV patterns, reduce collisions against darkened interiors (Klem & Saenger, 2013), although species may vary in their ability to detect this type of mitigation (Håstad & Ödeen, 2014). To round out our sample and to identify any additional buildings with frequent collisions, we selected three buildings from staff or ornithologist suggestions of potential high-collision buildings. These buildings had incidental reports of past collisions or large areas of reflective windows (Hager et al., 2013; Klem, 2008). Table 1 details the distribution of risks and collisions across the eight buildings, and describes the area and percentage of the monitored façade’s window cover ((i.e., window area/façade area)*100) and area and percentage of Feather Friendly^®^ treated mirror ((treated mirror area/total mirror area)*100).

**Table 1:** Building characteristics and collisions.

Winter	Building	Window area (m^2^)	Window cover (%)	Mirror area (m^2^)	Treated mirror (%)	Pear trees (0,1)	Mirrored windows (0,1)	Bird-friendly glass (0,1)	Collisions (total n)
1	3	144	26.99	119	0	1	1	0	15
2	3	144	26.99	119	30.89	1	0	0	10
2	4	431	39.41	–	–	1	0	0	9
2									
2	2	660	23.69	–	–	0	0	0	2
2									
2	5	788	40.59	–	–	1	0	0	0
2	6	3,342	73.78	–	–	0	0	0	0
2	7	1,219	90.05	–	–	0	0	0	0
2	1	1,860	24.97	–	–	0	0	1	0
2	8	1,002	30.40	–	–	0	0	1	1

**Notes.**

Winter 1 was November 9, 2017–January 2, 2018 and winter 2 was November 13, 2018–January 12, 2019. Window or mirror area refers to the window or mirror area on all monitored sides of the building. Monitoring included all four sides (for buildings 1, 2, 4, 5, and 6) or certain sides (for 3, 7, and 8) of buildings at the University of Utah, Salt Lake City, Utah, USA. Window cover represents the percent of the façade in windows; treated mirror represents the percent of Feather Friendly^®^ mitigated mirrored surface. Pear trees equal 1 when within 4.9 m of building. Bird-friendly glass had either permanent ceramic frits or ORNILUX UV-embedded patterns.

### Collision monitoring

Four monitors counted as collisions any stunned birds, fatalities, or feather piles (>12 feathers within 0.09 m^2^) found under windows and extending 3 m from the designated building sides. Monitors verified their building observations by taking time-stamped cellphone photos of each building they observed. We uploaded photos to a shared drive and recorded the presence or absence of a collision by building and date on a spreadsheet on a shared drive. One monitor evaluated all data completions weekly to ensure complete data collection. We double-bagged carcasses or feather piles and delivered them to an ornithology lab with an appropriate salvage license for research and disposal (Hager & Cosentino, 2014). We photographed all carcasses and stunned birds, where possible, and uploaded the images to an iNaturalist project.

Monitors received on-site training, which included detection of carcasses left outside the study buildings as a training exercise. Monitors also practiced logging data location and date on the spreadsheets, uploading building photos, and using iNaturalist. Although schedules permitted only one monitor in most cases, we assessed the interrater reliability of pairs of monitors for 82 building observations. In 61 cases, two monitors evaluated the same building at the same time, going in opposite routes. In 21 instances, a second monitor collected data at the same building but shortly after the first monitor finished, so that the first monitor was unaware that a second monitor was checking his/her work. Monitoring typically occurred during afternoon hours.

In order to monitor the mitigation building, it was important to define comparable pre-mitigation and post-mitigation winter monitoring periods. We defined winter 1 to start November 9, 2017, when the first collision alerted us to the problem, and to end January 2, 2018. This period was defined to capture the multiple winter-season fatalities that initially drew our attention. We verified that January 2 was an appropriate end of winter 1 by observing for 10 additional days, during which no collisions occurred. We defined winter 2 to start November 15, 2018, and end January 12, 2019. We again confirmed that no additional collisions occurred at the mitigation building for 10 days before and after winter 2. For both winter 1 and 2, we monitored for an average of 2.78 days per week, ranging from 1 to 5 observations per week or partial week across 9 weeks. The 2.78 average number of observations per week was consistent with a time interval found to prevent carcass removal by scavengers (Hager, Cosentino & McKay, 2012; Kummer et al., 2016a; Riding & Loss, 2018). In general, as is common for collision studies, we assume that we have undercounted the true number of collisions, given that some birds survive collisions and carcasses can be difficult to detect due to predation, landscaping, leaves, ground cover, or snow.

**Figure 2:**
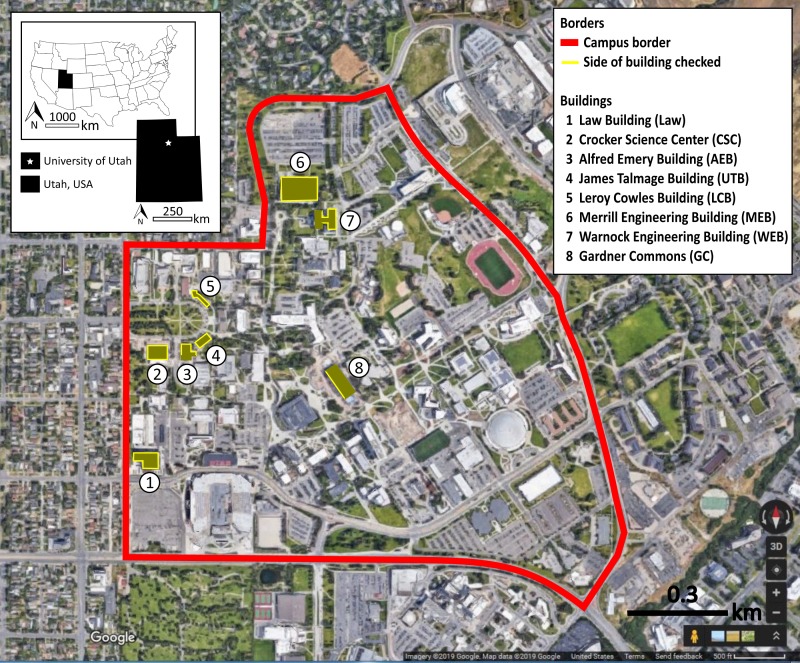
Map of the eight monitored buildings. The solid lines around each building indicate which sides were monitored for bird-window collisions. These buildings were monitored during November 13, 2018–January 12, 2019 at the University of Utah, Salt Lake City, Utah, USA. Map data ©2019 Google.

To evaluate the winter 2 sample of eight buildings for collisions, we monitored the buildings from November 13, 2018 to January 12, 2019. Schedules did not permit an equal number of observations per building. Across the eight buildings, we monitored 25 days for four buildings, 21 days for two buildings, and 17 days for two buildings. Figure 2 shows the eight buildings and notes the route taken around the building, with routes completely encircling five of the buildings.

### Outreach methods

In order to discover whether any other buildings had frequent noticeable collisions and to enhance campus awareness, we developed an iNaturalist project: University of Utah Bird Window Collision Project (iNaturalist.org). That project accepted reports of campus bird fatalities from all causes and with varying levels of documentation, to register the concern by those who reported sightings. The project included details of bird fatalities that were outside of the winter 1 or 2 monitoring periods for any of the monitored buildings, or that occurred by monitored buildings in front of a blank wall, not windows.

We also designed and distributed 20 posters in main campus buildings that attracted much visitor traffic (e.g., student union, library, major classroom buildings). The posters, displayed throughout data collection, detailed how campus users who witnessed bird strikes could contact us via phone, email, or post to iNaturalist. The co-authors also conducted media outreach to the faculty/staff newsletters, the student newspaper and a local birding listserv and social media page. In addition, we explained our activities when passers-by were curious about our activities, which included walking through bushes next to the buildings. The University of Utah institutional review board declared the research exempt from IRB review (IRB_00117279).

### Data analysis

To assess interrater reliability we used the intraclass correlation coefficient (ICC), which provides a score from 0 to 1.0, with 1.0 indicating perfect agreement (Shrout & Fleiss, 1979). The ICC 95% confidence interval values are interpreted as evidence of good reliability if values are between 0.75 and 0.90 and excellent reliability if values are above 0.90 (Koo & Li, 2016). We derived the ICC using a one-way random effects test, appropriate when different monitors contribute to reliability observations, and selected the value appropriate for reliance on a single rater’s score rather than an average score across monitors (Koo & Li, 2016). All analyses used IBM SPSS Statistics 25 software (IBM, Armonk, NY, USA).

To evaluate whether Feather Friendly^®^ bird-deterrent film decreased bird window collisions at the mitigation building, we used generalized estimating equations (GEE) with a Poisson distribution, an auto-regressive correlation structure across repeated measures, a log link function, and significance levels *p* <  0.05 (Ma, Mazumdar & Memtsoudis, 2012). This technique is useful for analysis of correlated count data (Norusis, 2008), which is typical for repeated counts of bird-window collisions, and it averages across weeks in the pretest and posttest times. The outcome variable is the count of bird-window collisions per week across the nine full or partial weeks of observation, for both winter 1 and winter 2. The factors tested include the between group factor of area (treatment and control areas, with mitigation applied to the treatment area between winters 1 and 2) and the within subjects factor of season (winter 1 and 2, monitored before and after the mitigation). If mitigation is effective, this type of pretest-posttest with untreated control group design can yield a significant area by season interaction, reflecting greater reduction of collisions in the mitigation area than in the untreated control area. The interaction test is followed by simple effects tests, using Wald *χ*^2^.

In winter 2 we tested whether collisions were related to the risk factors of the mirrored façade, pear tree proximity, and bird-friendly glass (ORNILUX or fritted glass), using GEE analyses as detailed above. All three predictor variables were scored as dummy variables (1 = present, 0 = absent). To reflect the uneven number of observations per building, we analyzed the data for each monitored day, not summed across weeks. To appreciate both the combined and direct effects of the three predictors, we present five models: all three predictors together, each predictor alone, and the best fit model. Note that the three predictors were not completely crossed factors in the current study, so that it was not possible to test their interaction terms (e.g., no buildings had mirrored windows without pear trees). We used the corrected quasi-likelihood under independence model criteria (QICC) goodness of fit test to compare models, which penalizes for model complexity and in which smaller values indicate better fit (Norusis, 2008). We also provide descriptions of the phi statistics that show relationships among the predictors.

## Results

### Interrater reliability

The intraclass correlation coefficient (ICC) for different monitors counting collisions at the same building at close to the same time was 0.93 (95% CI [0.90–0.96]), which indicates good agreement (Koo & Li, 2016; Shrout & Fleiss, 1979).

### Mitigation building collisions

During winter 1, prior to mitigation, the mitigation building had 15 bird-window collisions. One was a Mourning Dove (*Zenaida macroura*), two were American Robins (*Turdus migratorius*), and the rest were Cedar Waxwings (*Bombycilla cedrorum*). During winter 2, all 10 collisions were Cedar Waxwings. We observed two stunned birds that did not immediately die after a window collision.

### Feather friendly^®^ mitigation evaluation

**Figure 3:**
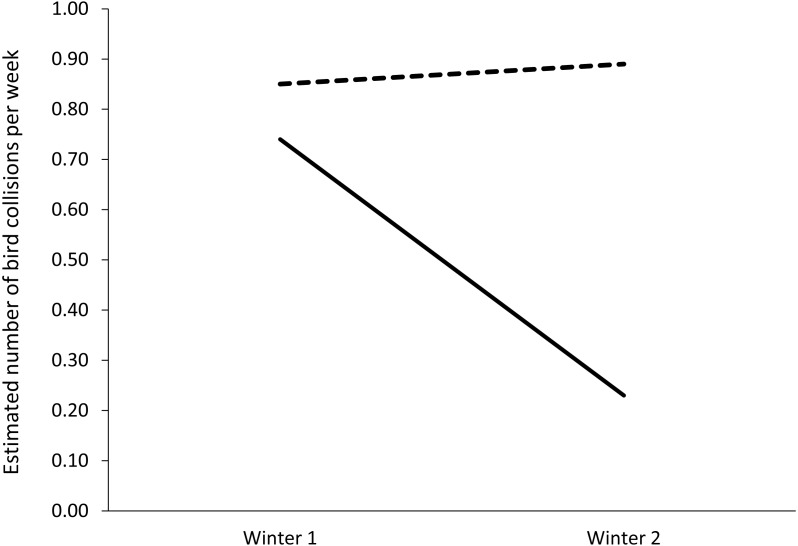
Bird collisions per week, pre- and post-mitigation: estimated marginal means from generalized estimating equations. Dashed line, Control area; undashed line, Treated area. The treated area had mitigation applied between winter 1 and 2; the control area did not. The data were gathered from one building at the University of Utah, Salt Lake City, Utah, USA. Winter 1 included November 9, 2017–January 2, 2018. Winter 2 included November 15, 2018–January 12, 2019.

Descriptively, the treatment area collisions declined from seven before mitigation to two after mitigation, a 71% reduction. The control area had eight collisions at both times. The generalized estimating equation demonstrated a significant area by season interaction, indicating different amounts of change for treatment and control area collisions (Wald *χ*^2^ = 9.14, *d.f.* = 3, *p* = 0.03, Fig. 3). The follow-up simple effects test showed that the treatment and control areas had similar numbers of collisions per week during winter 1, prior to mitigation (Wald *χ*^2^ = 0.08, *p* > 0.05). At winter 2, the mitigated area had significantly fewer collisions than the control area (Wald *χ*^2^ = 5.03, *d.f.* = 1, *p* = 0.03). The control area showed no significant change from winter 1 to 2 (Wald *χ*^2^ = 0.02, *d.f.* = 1, *p* > 0.05) and the treatment area showed a trend toward a decline (Wald *χ*^2^ = 3.45, *d.f.* = 1, *p* = .06). This pattern of results supports the effectiveness of mitigation.

### Risk factors, winter 2

In addition to the 10 Cedar Waxwing collisions at the mitigation building during winter 2, there were eight Cedar Waxwing collisions and one feather pile at the adjacent eastern building 4, one Cedar Waxwing and one feather pile at building 2, and one feather pile at building 8.

When all three predictors were in the model, pear tree proximity explained a significant amount of unique variance, (*p* = 0.04, QICC = 95.89, Table 2). In this small sample of eight buildings, risk factors were somewhat correlated (e.g., pear trees and mirrored windows, Phi = 0.46; pear trees and bird-friendly glass, Phi = − 0.50; both *p* <  0.01). Models 2 through 4 show how these predictors fare as direct predictors by including each one alone. Model 2 shows that a building near fruiting pear trees, with an exponentiated B (Exp(B)) or odds ratio of 8.54, increased the odds of collision by 8.54, compared to buildings far from fruiting pear trees (QICC = 94.96). Similarly, a building with mirrored windows had 5.04 greater odds of a collision than for buildings without mirrored windows (QICC = 100.79). The best-fit Model 5 included pear trees and mirrored windows (QICC = 93.97). Although the bird-friendly glass did not achieve statistical significance, only one collision occurred at the building with fritted windows and none at the building with ORNILUX glass (QICC = 107.00). The goodness of fit comparisons show that the best-fit model is similar to (within two QICC points of) the pear tree-only and full 3-predictor models.

**Table 2:** Bird-window collisions per day predicted by building risk and protective features: generalized estimating equation results. Data were collected in winter season 2018–2019 at the University of Utah, Salt Lake City, Utah, USA.

					95% Wald C.I.	
	B	S.E.	*p*	Exp(B)	Lower	Upper
Model 1: all three predictors						
Intercept	−3.39	0.73	0.01	0.03	0.01	0.14
Pear trees	1.67	0.81	0.04	5.31	1.08	26.05
Mirrored windows	0.80	0.48	0.09	2.22	0.87	5.66
Bird-friendly glass	−0.35	1.27	0.78	0.70	0.06	8.48
Model 2: Pear tree only						
Intercept	−3.52	0.61	0.01	0.03	0.01	0.10
Pear trees	2.14	0.65	0.01	8.54	2.38	30.68
Model 3: Mirrored windows only						
Intercept	−2.55	0.33	0.01	0.08	0.04	0.15
Mirrored windows	1.62	0.49	0.01	5.04	1.93	13.21
Model 4: Bird-friendly glass						
Intercept	−1.87	0.25	0.01	0.15	0.09	0.25
Bird-friendly glass	−1.89	1.19	0.11	0.15	0.02	1.56
Model 5: Pear + mirrored, best fit						
Intercept	−3.52	0.60	0.01	0.03	0.01	0.10
Pear trees	1.80	0.69	0.01	6.06	1.57	23.49
Mirrored windows	0.80	0.48	0.09	2.22	0.87	5.65

**Notes.**

B B coefficient S.E. Standard error of B Exp(B) odds ratio CI confidence interval

### Citizen outreach and ad-hoc reports of bird fatalities

Our encouragement of ad hoc reports, including all seasons and places on campus, led to 22 additional reports, including 19 collision fatalities and three non-collision fatalities. Collisions included multiple reports of Cedar Waxwings (*n* = 4), pigeons and doves (likely *Columba livia* and/or *Zenaida macroura*, *n* = 3), and hummingbirds (likely Broad-tailed (*Selasphorus platycercus*) or Rufous (*Selasphorus rufus*), *n* = 2). They also included single reports of House Finch (*Haemorhous mexicanus*), Lesser Goldfinch (*Spinus psaltria*), Mourning Dove, American Robin, unknown carcass, Blue-gray Gnatcatcher (*Polioptila caerulea*), Northern Flicker (*Colaptes auratus*), Hermit Thrush (*Catharus guttatus* ), and Ruffed Grouse (*Bonasa umbellus*). The latter two surprised us, given few eBird listings on campus for Hermit Thrushes and none for Ruffed Grouse; the thrush collided with another mirrored surface on campus. The non-collision fatalities included carcasses found away from windows for two Great Horned Owls (*Bubo virginianus,* confirmed by local eBird authority Frisch, personal communication, November 18, 2018). It appeared that a Black-billed Magpie (*Pica hudsonia*) had been trapped by bird-deterrent netting in a parking garage, a surprising new source of danger to birds. Sixteen of the 22 reports were from among the eight buildings we monitored, but outside of winter 1 and 2 or found beside solid walls rather than windows.

## Discussion

### Mitigation study

Understanding the risk factors underlying bird window collisions and the efficacy of mitigation strategies can facilitate efforts to reduce their fatalities. We found that applying Feather Friendly^®^ film, which left small visible white dots on the exterior of a mirrored surface, decreased bird-window collisions by 71%. Control windows, with no mitigation, had the same number of collisions over time. The significant area by season interaction and the pattern of results enhances our confidence that the mitigation itself caused fewer collisions, rather than some naturally occurring change.

Other mitigation studies have demonstrated reduced collisions, but missed key aspects of study design and/or analysis needed to attribute reductions to the mitigation. The only study we found that discussed a control area was a technical report from a Feather Friendly^®^ mitigation at the University of Pennsylvania (Durrance, 2015). Fatalities declined there by 100% for mitigation and 75% for control areas. This difference was not statistically tested, and pretest and posttest seasons differed, limiting our ability to attribute declines to mitigation alone. The sharp reduction in collisions from the control area suggested that seasonal differences from pretest to posttest or some other uncontrolled factor was causing some of the decline. At Duke University, USA, after applying Feather Friendly^®^, fatalities declined by 88% in spring and 53% in fall (Winton, Ocampo-Peñuela & Cagle, 2018). This mitigation covered a glass walkway and glass towers, which may have made it difficult to find suitable control areas elsewhere on campus and, due to a small sample, there was no statistical test of effectiveness. At the Federal University of São Carlos, Brazil, after mitigation with sparsely spaced (about 0.25 per m^2^) bird of prey silhouettes there was a 47% decline in carcasses, but the reduction was not statistically significant (Brisque, Campos-Silva & Piratelli, 2017). In contrast, after more densely spaced (0.41 per m^2^ ) UV decals were placed on five of 15 windows on a house on a nature preserve in Colombia, the treated windows had a significant 84% decline, but there was no test of untreated windows (Ocampo-Peñuela, Peñuela Recio & Ocampo-Durán, 2016a). Other reports of mitigation seemed promising, but were also missing control groups and other key details (De Groot, 2018; Elmhurst & Grady, 2017).

### Risk factor study

At winter 2 we expanded observations to eight buildings and found that nearby fruiting pear trees and mirrored surfaces were related to more collisions. Our results were similar to other studies that have found more collisions with windows near tall trees or that reflect trees (Borden et al., 2010; Gelb & Delacretaz, 2006; Klem et al., 2009; Kummer, Bayne & Machtans, 2016b). The pear trees in our study were tall enough to face the upper stories of our 3-story buildings, reflected in windows or mirrors, and provided the additional lure of edible pears. Mirrored surfaces have not been widely studied, although two technical reports provided anecdotal reports of high risks associated with mirrored surfaces (Evans Ogden, 2002; Sheppard & Phillips, 2015) and one article noted about 29 carcasses per mirrored building across a year (O’Connell, 2001). Sheppard & Phillips (2015) suggest that mirrored surfaces are especially risky because they reflect at all hours of the day and their reflections can be of trees or open sky, both of which signal places to fly. Although bird-friendly ceramic frit or ORNILUX glass did not emerge as a significant predictor of reduced risk, there was only one fatality across the two buildings with bird-friendly glass. Some of the comparison buildings without bird-friendly glass had no winter collisions but had collisions in other seasons; this suggests that monitoring across more seasons might yield significant differences for bird-friendly glass.

### Winter season effects

More published studies focus on migratory than winter season collisions (Loss et al., 2014), when total collisions are fewer (Borden et al., 2010; Hager et al., 2008; Kummer, Bayne & Machtans, 2016b; Schneider et al., 2018). Our results revealed greater Cedar Waxwing collision numbers and percentages than found in other studies. We found waxwings accounted for 91% of identifiable collisions (30 of 33, excluding the three feather piles, Table 1). The greatest percentages of Cedar Waxwings among identifiable collisions from other studies included 11 of 70 (15.7%) in North Carolina (Ocampo-Peñuela et al., 2016b) and seven of 50 (14%) in Edmonton, Alberta (Kummer & Bayne, 2015). Other studies recorded even fewer Cedar Waxwing collisions: 14 of 415 and zero of 13 (Hager et al., 2008), zero of 16 (Table 1, Hager & Craig, 2014), zero of 116 (O’Connell, 2001), one of 20 (Somerlot, 2003), 1 of 271 (Borden et al., 2010), 3 of 108 (Bracey et al., 2016), zero of 46 (Hager et al., 2013), and 10 of 945 (Dunn, 1993). Nevertheless, in a systematic review of 23 past studies, Cedar Waxwings were the fifth most vulnerable species to collision, with 3.6 times the risk of collision as an average species (Loss et al., 2014). Thus, Cedar Waxwings collisions are present in other studies, but with fewer percentages and numbers of collisions than in this study.

Our high numbers of Cedar Waxwing collisions are consistent with the fact that our two highest collision buildings faced a row of fruiting pear trees, an important winter source of food (Witmer, 1996; Witmer, Mountjoy & Elliot, 2014), and had mirrored and/or reflective windows. The mitigation building, with 22 waxwing collisions in winters 1 and 2, had mirrored windows, reflective windows, and nearby pear trees. The adjacent eastern building, with eight waxwing collisions in winter 2, had the longest façade of reflective windows facing a line of pear trees. Cedar Waxwings triple their food intake in colder temperatures (−21 °C) compared to warmer temperatures (21 °C) (McWilliams, Caviedes-Vidal & Karasov, 1999). Furthermore, birds that glean in tree canopies are more susceptible to collisions (Wittig et al., 2017). The plentiful food in pear trees and extra winter feeding time extends waxwing exposure to collision dangers when laden trees are near reflective or mirrored windows. This effect is likely to occur with other fruiting trees that draw winter birds. Our casual observations during monitoring suggested that the numerous pear trees attracted more waxwings than the few crabapple trees. Perhaps some combination of fruit qualities–amount, ripeness, or taste—drew waxwings to pear trees, a suggestion that requires further testing. We wondered whether fruiting trees planted very close to windows could reduce collision mortality. When bird feeders are less than 1 m from windows, collisions from less than a 1 m launch often have insufficient momentum to cause fatalities (Klem et al., 2004). However, our grounds crew said that they avoid planting trees next to buildings due to extra upkeep and potential damage from roots.

### Challenges of mitigation

It is easier to count bird carcasses than to prevent their deaths, which may explain why there are fewer studies of mitigation than of carcass collection. Drawing from our experience, we suggest the need for more research on mitigation efficacy and implementation challenges (Ocampo-Peñuela et al., 2016b) and more action ecology approaches to enact collision prevention policies (White et al., 2015). Toward that end, we share our perceptions of the major mitigation barriers: lack of awareness and concern, proliferation of risk factors including fruiting trees and glass, limited mitigation funding, complications of implementation, and ethical considerations (Schneider et al., 2018).

Bird-window collision deaths often go unnoticed (Bracey et al., 2016). To counter invisibility of the problem, we used vivid communications in our posters, iNaturalist project, and presentations to reporters and staff. Vivid messages can foster attention, cognitive and affective processing, memorability, and persuasiveness of a message (Bator & Cialdini, 2000; Blondé & Girandola, 2016). Thus, presenting images of dead birds, referring to the mitigation building as a “hotspot of death,” and titling a presentation to an architectural research organization “Designed for Death” all appeared to facilitate audience engagement. Klem (2014) has also used vivid messages, stating that 100 million window collision fatalities are equivalent to 333 Exxon Valdez oil spill bird fatalities. In addition, institutional “green” identities can be important (Lozano et al., 2015), so vivid threats to such identities may be useful. For example, our outreach materials compared our “hotspot of death” to the well-known US Bank (Viking) Stadium in Minneapolis. Bird advocates had warned of the dangers of its 18,581 m^2^ of glass. Subsequent bird fatalities led to dozens of negative media reports (e.g., Peters, 2018). We computed that the stadium had 0.40 fatalities/100 m^2^ glass (Bahls, 2017) compared to our mitigation area having 20.80/100 m^2^ pre-mitigation and 5.94/100 m^2^ post-mitigation collisions. We created bar charts from these averages to share with university staff. The charts portrayed a stark difference, demonstrating that the mirrored façade, though small, was deadlier per area than the well-known stadium site. We need systematic research to test how effective these vivid and identity-relevant campaigns can be to create resources for mitigation.

Fruiting trees and glass, two key ingredients creating risks for birds, are increasing in the US. Fruiting trees are becoming more popular, with flowering pears the most popular trees in some of the newer local suburbs around Salt Lake City (Avolio et al., 2018) and popular to the point of invasiveness elsewhere in the US (Culley & Hardiman, 2007). As new construction advances, the amount of glass in buildings has been increasing (Pariafsai, 2016), and technologies that improve the energy efficiency of glass will make glass buildings even more popular (Arbab & Finley, 2010). As the current research shows, mirrored glass poses extra risks to birds. The irony is that internet architectural sites often promote mirrored buildings as nature-friendly. The writers praise mirrored surfaces because they “dissolve into the woods,” connect to the verdant landscape (McKnight, 2018), or create a natural experience (Johnson, 2015).

Although the prevention of bird-window collisions is increasingly recognized in building design guidelines (City of Toronto, 2016; Sheppard & Phillips, 2015; U.S. Green Building Council, 2019b), guidelines do not provide quick and easy solutions. Guidelines are typically voluntary. The most popular system, the Leadership in Energy and Environmental Design (LEED), requires a certain number of points from a range of options to certify a new building as green. The point system appears to incentivize energy efficiency and other green goals over biodiversity (Ogden, 2014). For example, one point is awarded for avoiding bird collisions (pilot credit 55) by requiring buildings to limit reflective glass to 15% of surfaces up to 36 ft high and mandating three years of monitoring (U.S. Green Building Council, 2019b). However, four points are available from daylighting 75% of the floor area (3 daylighting points) and providing unobstructed exterior views (e.g., no frits or patterned glazing) to birds or fruiting trees at least 7.5 m away (1 quality view point, U.S. Green Building Council, 2019a). Perhaps not surprisingly, LEED-certified buildings can have large numbers of bird-window collisions (Kahle, Flannery & Dumbacher, 2016; Ocampo-Peñuela et al., 2016b; Ogden, 2014). Researchers have noted that the point system could be improved to address the dangers of planting trees that bring birds near reflective windows (Kensek, Ding & Longcore, 2016). Finally, there is no point system to incentivize mitigation of existing buildings. Unless collision research starts to inform prevention and alter architectural or landscaping preferences and certification, there will be growing numbers of buildings to mitigate.

More description of mitigation funding methods and sources would help researchers plan their efforts. After an administrative refusal to fund our work, we received a $10,000 Sustainable Campus Initiative Fund (SCIF) grant, supported by student fees. Over 200 universities have green funds (AASHE, 2019), but we only found two reports of colleges using these fees for mitigation studies: William and Mary (Weber, 2016) and University of Pennsylvania (Durrance, 2015). At University of Pennsylvania, the initial mitigation cost was $22,000 ($9,000 materials, $8,500 labor, and $3,000 lift rental; Durrance, 2015) and some follow-up work has also been funded there (Simonton, 2018). Some universities provided funding after student government resolutions (Ocampo-Peñuela et al., 2016b) and student petitions and an artwork installation of folded cranes representing dead birds (Sanchez, 2018). We encourage other researchers to share funding sources and methods.

Implementing bird collision mitigation can be complicated (T O’Connell, pers. comm., 2019). Altering the exteriors of windows requires special permission from officials. Occupants of some facilities consider clear views through windows to be more important than mitigation. On our campus, mitigating buildings within a historic district requires approval from a historic architect. Applying for funding can take months. At a Virginia Polytechnic Institute and State University site, despite research pinpointing the two deadliest buildings and despite a blog documenting fatalities, administrators refused to mitigate (Qaiser, 2019; Schneider et al., 2018).

Finally, the ethics of having control areas to evaluate mitigation deserve consideration. If our budget had allowed, we would have mitigated the entire mirrored façade. The limited budget led to a stronger research design, but we understand why researchers may choose to mitigate all problem areas at once. However, we also believe that knowledge advances through strong studies with control groups. We suspect it is likely that mitigation funding often comes in phases. If so, current control areas could become future mitigation areas. Given the costs of mitigation, advocates always need to consider whether scarce resources should go to mitigation or other needs. We believe that by raising awareness and campus concern, the non-monetized educational benefits also add value to mitigation.

## Conclusion

Our research demonstrated that mirrored surfaces and proximity to fruiting pear trees posed particular risks to Cedar Waxwings wintering at the University of Utah. We exploited a rare opportunity for a pretest-posttest study with a control group, which might serve as a strong research design others can consider. Feather Friendly^®^ mitigation was effective and we encourage future evaluations to establish its efficacy across other circumstances and with larger samples. Our study of risk factors led us to appreciate that society has created a deadly convergence of factors that put birds at risk. As increasing amounts of glass are incorporated into new buildings and fruit trees near buildings proliferate, bird-window collisions are a call to action. We encourage researchers to share details of funding and techniques that facilitated their mitigation efforts. Our results support several policy actions: mitigating the worst sites, banning mirrored building construction, keeping fruiting trees away from dangerous windows, and adopting bird-friendly landscaping and design policies.

##  Supplemental Information

10.7717/peerj.7620/supp-1 Data S1 Raw data

## Additional Information and Declarations

### Data Availability

The following information was supplied regarding data availability: The raw data is available as a Supplemental File.
